# Life’s simple 7 and cardiovascular disease risk knowledge in Hong Kong

**DOI:** 10.1186/s12872-019-1171-7

**Published:** 2019-08-02

**Authors:** Vivian W. Y. Lee, Felix Y. H. Fong, Franco W. T. Cheng, Bryan P. Y. Yan

**Affiliations:** 1 Center for Learning Enhancement and Research, The Chinese University of Hong Kong, Shatin, NT Hong Kong; 2 School of Pharmacy, Faculty of Medicine, The Chinese University of Hong Kong, Shatin, NT Hong Kong; 3 Department of Medicine and Therapeutics, Faculty of Medicine, The Chinese University of Hong Kong, Shatin, NT Hong Kong

**Keywords:** Preventive cardiology, CV health, Epidemiology

## Abstract

**Background:**

This study aimed at investigating the CV health and CV disease knowledge in terms of LS7 score among 3 age groups in Hong Kong.

**Methods:**

A cross-sectional multicenter observational study was conducted to observe the CV health and disease risk knowledge in Hong Kong. Elderly subjects were recruited from 15 elderly centers, whereas young adults and the middle-aged were recruited from 6 on-campus health check sessions. Subjects’ demographics, lifestyle behavior and risk knowledge were obtained through questionnaire while their body mass index, random capillary blood glucose, blood cholesterol and blood pressure were measured. LS7 score and risk knowledge score was calculated.

**Results:**

The LS7 of younger adult, middle-aged and elderly were 10.6 ± 1.3, 9.3 ± 1.9 and 9.7 ± 1.7 respectively. Only 0.6% participants have attained ideal CV health and 35.9% have 5 to 7 ideal CV health metrics. Elderly performed worst in risk knowledge with a score of 8.1 ± 3.3 while young adult and middle-aged were similar (9.6 ± 1.8 and 9.7 ± 1.5). 71% of the participants correctly identified ≥9 components. Logistic regression revealed that subjects aged ≤65 years (OR 2.341, 95% CI 1.779 to 3.080) and with tertiary education (OR 2.031, 95% CI 1.527 to 2.701) were more likely to obtain optimum LS7. No association was found between having optimum LS7 and full knowledge.

**Conclusion:**

Only few adults in this study population had ideal CV health as defined by AHA. Knowledge has no association but young age and tertiary education has positive association with CV health.

**Electronic supplementary material:**

The online version of this article (10.1186/s12872-019-1171-7) contains supplementary material, which is available to authorized users.

## Background

Cardiovascular diseases (CVD) are one of the most burdensome diseases with high mortality. According to the World Health Organization (WHO) Non-communicable Diseases Country Profiles 2018, CVD were the first leading cause of death globally claiming 17.9 million lives in 2016, which is equivalent to 31% of the total deaths worldwide [1]. On the other hand, the burden of CVD in terms of medical cost is also huge in many countries. In the European Union, the overall expenditure on CVD in 2015 was estimated to be €210 billion (HK$2.16 trillion, €1 = HK$10.3), in which only 53% was direct medical cost [2]. The other half came from indirect costs such as productivity loss and informal care, which revealed the profound effect of long-term management of CVD. In the United States, the estimated economic cost of CVD in 2015 was approximately US$555 billion (HK$4.33 trillion, US$1 = HK$7.8), which was the highest among all disease states [3]. In Hong Kong, CVD was also one of the top three leading causes of death in 2018 accounting for 12.8% of all-cause deaths [4, 5]. CVD were also one of the top five disease groups with the highest number of in-patient discharges and deaths in all hospitals, and the increasing trend was still on-going [5, 6]. Figures from the Hospital Authority revealed that diseases of circulatory system occupied the largest proportion of the total health expenditure [7]. These statistics indicate that preventing CVD could significantly reduce the health expenditure.

The American Heart Association (AHA) issued guidelines on the prevention of CVD back in 2002 and updated in 2019. Healthy lifestyle such as sufficient physical activity and balanced dietary intake is recommended to reduce the risk of CVD [8]. In 2010, AHA set up the 2020 Impact Goals aiming at improving the cardiovascular (CV) health of all Americans by 20% while reducing deaths from CVD and stroke by 20% by 2020 [9]. Seven behaviors and health factors of individuals were identified as the keys to acquire ideal CV health and primordial prevention of CVD events. These seven metrics, or known as Life’s Simple 7 (LS7), include the assessment of smoking status, physical activity, dietary intake, body weight, blood pressure, blood glucose and cholesterol. Multiple studies have assimilated these 7 health aspects into CVD risk estimation and the results were promising [10, 11].

Besides, different CVD risk factors have been identified for decades. Sufficient knowledge is necessary to motivate an individual to change his behaviour [12]. Patients with more extensive knowledge about CVD risk factors were found to have better prevention-seeking behaviour [13]. Foreign studies have used a variety of methods to examine the knowledge about CVD risk factors [14–21]. Diverse results were obtained with respect to different populations. The CARDIA study showed that young adults had low knowledge level about CVD risk factors and knowledge level does not predict 10-year change in risk factor [16]. In a Canadian study, the knowledge level among the elderly was found to be consistently lower than the younger age group [17]. In a Croatian study, it was found that about one-fourth of high CVD risk elderly failed to recognize their own risk [22]. Education level [18], income level [19] and ethnicity [20, 21] were found to influence CVD risk knowledge. However, there is a lack of data about the understanding and awareness of CVD among Hong Kong people.

This project aimed at investigating the prevalence of Hong Kong adults who achieve ideal CV health and thus adding value to worldwide LS7 comparisons and bridges the gap and assesses the CV health and CVD risk knowledge of Hong Kong people.

## Methods

### Study design

This was a cross-sectional multicenter observational study to assess the CV health and CVD risk factor knowledge in Hong Kong in 2013. The current study was approved by the research ethics committee of Joint Chinese University of Hong Kong-New Territories East Cluster.

### Setting

A total of 22 health check sessions were held in 15 non-governmental elderly centers between July and August 2013 and the campus of Chinese University of Hong Kong in November 2013. Each health check session was divided into two sections, namely health assessment with questionnaire and critical health markers measurement which includes blood pressure, blood glucose, cholesterol and body mass index [9].

Blood pressure was taken using Ormon HEM-7113 Automatic Blood Pressure Monitor (Ormon Healthcare, Kyoto, Japan) after resting in a seated position for at least 5 min. Blood glucose level was measured by BREEZE® 2 Blood Glucose Monitoring System (Bayer, Leverkusen, Germany) with documented time post-prandial. Blood cholesterol level, including LDL, HDL, TG and total cholesterol, were measured by CardioChek® PA (Polymer Technology Systems, Indianapolis, USA) with PTS Lipid Panel Test strip. Each blood sample was collected by a lancet with 40 μL capillary blood collector.

### Participants

Elderly subjects (older than 65 years old) were enrolled from the above-mentioned elderly centers while younger (18 to 39 years old) and middle-aged (40 to 65 years old) adults were recruited in the campus of Chinese University of Hong Kong.

All recruitment was conducted on a voluntary basis. Subjects would be included if they were able to complete both the questionnaires and critical health marker measurements. Subjects with poor prognosis with life expectancy less than 12 months undergoing other clinical trials were excluded from the study.

### Outcome measurements

The questionnaire for health assessment was designed to collect demographic data, CVD awareness and knowledge, CVD risk-lowering action and lifestyle background (see Additional file 1). These data, together with the critical health markers was then converted into LS7 score, which is the primary outcome of the current study. LS7 is a scoring system proposed by AHA and takes into account of 7 health aspects: smoking status, physical activity, diet, BMI, blood glucose, blood cholesterol and blood pressure. Each aspect was rated at “ideal”, “intermediate” or “poor”, corresponding to a score of 2, 1, or 0 respectively [23]. Individuals obtaining 14 points were defined to have ideal CV health. Table 1 summarizes the AHA LS7 health categories.

**Table 1:** American Heart Association Life’s Simple 7 Health Categories

Health Metrics	Ideal	Intermediate	Poor
Body Mass Index	< 25 kg/m^2^	25–29.9 kg/m^2^	≥30 kg/m^2^
Diet	0–1 Component	2–3 Components	4–5 Components
Physical Activity	≥150 min/week moderate exercise	1–149 min/week moderate exercise	None
Smoking	Never or quit > 12 months	Former ≤12 months	Current
Blood Pressure	SBP < 120 and DBP < 80 mmHg	SBP 120–139 or DBP 80–89 mmHg	SBP ≥ 140 orDBP ≥ 90 mmHg
Total Cholesterol	< 200 mg/dL	200–239 mg/dL	≥ 240 mg/dL
Blood Glucose	< 100 mg/dL	100–125 mg/dL	≥ 126 mg/dL

The secondary outcome measurement was CVD risk knowledge that was assessed in the “awareness and knowledge” section of the questionnaire. There were 19 types of health condition or behavior listed and subjects were required to determine whether each of them was a CVD risk factor by means of the three-point Likert scale, which includes the options ‘agree’, ‘neutral’ and ‘disagree’. Eleven of them, namely hypertension, diabetes mellitus, hyperlipidemia, smoking, lack of exercise, overweight, high salt consumption, high sugar consumption, advanced age, family history and stress, are true risk factors. Knowledge scores were calculated by summing up the number of correct identifications made. Obtaining a maximum score of 11 indicated a full knowledge on CVD risk factors.

### Statistical analysis

The results of continuous variables were presented as means ± SDs while that of categorical variables were presented as frequencies and percentage. LS7 scores were categorized according to the LS7 scoring system, with 0–4 being classified as inadequate, 5–9 as average, 10–14 as optimum CV health, and 14 as ideal CV health. CVD risk knowledge levels were dichotomized into “full knowledge of 11 components” and “insufficient knowledge”. Chi-square test was employed to test the differences of LS7 and CVD risk knowledge among three age groups, with a *p* value of less than 0.05 considered to be statistically significant. Logistic regression analysis was performed to estimate the effect of gender, age, education, birthplace and co-morbidity on LS7. All analyses were performed using SPSS version 20 (International Business Machines Corp., Armonk, New York).

## Results

### Baseline characteristics

Of the 1538 participating subjects, 996 (64.8%) of them had completed the whole questionnaire and critical health markers with valid results. The demographic characteristics were summarized in Table 2. The mean age of the studied subjects was 56.3 ± 25.5 years, and the mean age was 24.5 ± 5.4 years, 49.5 ± 5.4 years and 77.6 ± 6.9 years for young adults, middle-aged and elderly respectively. The number of participants in the 3 age groups were not balanced and three-quarters of them are female. There were also a higher proportion of participants with tertiary education.

**Table 2:** Demographic Data of Participants

Characteristics	Number	Percent
Total Number of Interviewees	996	100
Age		
18–39 (Younger Adult)	350	35.1
40–65 (Middle-aged)	94	9.4
> 65 (Elderly)	552	55.4
Male	279	28.0
Education		
Never	229	23.0
Primary Level	207	20.8
Secondary Level	151	15.2
Tertiary Level of Higher	374	37.6
Birthplace		
Hong Kong	426	42.8
China	504	50.6
Other Asian countries	31	3.1
Others	18	1.8
Self-reported Past Medical History (Multiple Responses)		
Hypertension	361	36.2
Diabetes Mellitus	141	14.2
Hyperlipidemia	212	21.3
Heart Disease	94	9.4

### Life’s simple 7 score

The LS7 distribution of the study population was presented in Table 3. The mean LS7 score was 10.0 ± 1.7. Among the three age groups, younger adult obtained the highest mean LS7 score of 10.6 ± 1.3, followed by the elderly (9.7 ± 1.7) and the middle-aged (9.3 ± 1.9). Overall, participants performed best in smoking status (93% ideal), followed by cholesterol (81% ideal) and BMI (74% ideal). The worst CV health metrics were blood glucose (39% ideal) and blood pressure (33% ideal), with over 20% of respondents having ‘poor’ performance (23% for blood glucose and 35% for blood pressure). The result of individual component varies in different age groups. Smoking status remained the most ideal metric achieved across all 3 age groups. Diet was the least ideal metric in young adults, physical exercise in the middle-aged group and blood pressure in the elderly.

**Table 3:** Results of Life’s Simple 7

	Young Adults	Middle Aged	Elderly	Total
Health Metrics				
BMI				
Poor	1.4%	1.1%	6.3%	4.1%
Intermediate	5.1%	19.1%	33.5%	22.2%
Ideal	93.4%	79.8%	60.1%	73.7%
Diet				
Poor	34.0%	27.7%	2.0%	15.7%
Intermediate	53.7%	55.3%	33.7%	42.8%
Ideal	12.3%	17.0%	64.3%	41.6%
Physical Activity				
Poor	19.4%	34.0%	10.1%	15.7%
Intermediate	71.4%	47.9%	19.9%	40.7%
Ideal	9.1%	18.1%	69.9%	43.7%
Smoking				
Poor	0.6%	3.2%	10.9%	6.5%
Intermediate	0.0%	0.0%	1.6%	0.9%
Ideal	99.4%	96.8%	87.5%	92.6%
Blood Pressure				
Poor	5.1%	25.5%	55.4%	34.9%
Intermediate	22.6%	45.7%	36.2%	32.3%
Ideal	72.3%	28.7%	8.3%	32.7%
Total Cholesterol				
Poor	2.3%	13.8%	5.1%	4.9%
Intermediate	7.4%	25.5%	16.7%	14.3%
Ideal	90.3%	60.6%	78.3%	80.8%
Blood Glucose				
Poor	10.9%	12.8%	32.8%	23.2%
Intermediate	35.7%	38.3%	38.2%	37.3%
Ideal	53.4%	48.9%	29.0%	39.5%
Overall Score				
Inadequate (0–4)	0.0 %	1.1%	0.0%	0.1%
Average (5–9)	18.9 %	43.6%	42.9%	34.5%
Optimum (10–14)	81.1%	55.3%	57.1%	65.4%
Total No. of Ideal Health Metrics Present				
7	0.6%	0.0%	0.7%	0.6%
6	6.0%	2.1%	8.2%	6.8%
5	36.6%	19.1%	25.0%	28.5%
4	40.6%	34.0%	32.6%	35.5%
3	12.6%	24.5%	22.5%	19.2%
2	3.7%	11.7%	9.6%	7.7%
1	0.0%	8.5%	1.4%	1.6%
0	0.0%	0.0%	0.0%	0.0%

The prevalence of ideal CV health was also summarized in Table 3. We found that only 0.6% (*n* = 6) of the 996 participants attained ideal CV health (ideal for all 7 health metrics), and 35.9% have 5 to 7 ideal CV health metrics. Out of the 6 participants, four were elderly and two were yound adults.

### CVD risk factor knowledge

Table 4 revealed the awareness of CVD risk factors in the study population. Of the 996 participants, 32.3% (*n* = 322) obtained a score of 11, i.e. correctly identified all 11 CVD risk factors. 71.0% (*n* = 708) of the participants correctly identified ≥9 components. Hypertension was the most well-recognized CVD risk factor (87.6%, *n* = 873) while diabetes mellitus and high sugar consumption had the lowest recognition (71.9%, *n* = 716 and 72.6%, *n* = 723). The mean CVD risk knowledge score was 8.8 ± 2.8. Among the three age groups, elderly performed worst with a mean score of 8.1 ± 3.3. The performance of young adult and middle-aged were similar with a mean score of 9.6 ± 1.8 and 9.7 ± 1.5 respectively.

**Table 4:** CVD Risk Factors Awareness

	Young Adults	Middle Aged	Elderly	Total
Risk Factors being Identified				
Age > 65 years	89.1%	80.9%	70.1%	77.8%
Hypertension	96.3%	97.9%	80.4%	87.7%
Diabetes Mellitus	75.1%	78.7%	68.7%	71.9%
Hyperlipidemia	94.9%	94.7%	74.5%	83.5%
Smoking	86.6%	90.4%	76.3%	81.2%
Lack of Exercise	86.3%	85.1%	77.4%	81.2%
Family History	92.9%	86.2%	55.6%	71.6%
High Salt Intake	86.9%	91.5%	78.8%	82.8%
High Sugar Intake	79.1%	74.5%	68.1%	72.6%
Stress	81.4%	89.4%	78.3%	80.4%
Overweight	91.4%	96.8%	78.8%	84.9%
Total No. of Risk Factors Identified				
11	40.0%	34.0%	27.2%	32.3%
10	24.3%	29.8%	20.3%	22.6%
9	17.1%	22.3%	14.5%	16.2%
8	8.3%	5.3%	8.7%	8.2%
7	4.0%	3.2%	4.7%	4.3%
6	3.1%	2.1%	4.9%	4.0%
5	0.6%	2.1%	3.6%	2.4%
4	0.6%	1.1%	2.9%	1.9%
3	1.1%	0.0%	2.5%	1.8%
2	0.6%	0.0%	1.6%	1.1%
1	0.0%	0.0%	2.2%	1.2%
0	0.3%	0.0%	6.9%	3.9%

### Predictors of cardiovascular health

The predictors of ideal CV health were shown in Table 5. Subjects with age equal or under 65 (OR 2.341, 95% CI 1.779 to 3.080) and subjects with tertiary education or above (OR 2.031, 95% CI 1.527 to 2.701) were more likely to have optimal LS7 (10–14 marks). Subjects born in Hong Kong were also more likely to have optimal LS7 (OR 1.514, 95% CI 1.155 to 1.984, *p* = 0.015). Male subjects, on the other hand, were less likely to have optimal LS7 (OR 0.402, 95% CI 0.306 to 0.529, *p* = 0.025). Subjects with history of hypertension (OR 0.440, 95% CI 0.337 to 0.575, *p* < 0.001), diabetes mellitus (OR 0.366, 95% CI 0.255 to 0.526, p < 0.001), hyperlipidemia (OR 0.477, 95% CI 0.349 to 0.650, p < 0.001) were also less likely to have optimal LS7 but no association was found for patients with history of heart disease in the current study. No association was seen between full knowledge and optimum LS7.

**Table 5:** Predictors of optimum Life’s Simple 7 Score

Characteristics	OR	95% CI
Age (≤65 years old)^a^	2.341	1.779–3.080
Education (Tertiary or above)^a^	2.031	1.527–2.701
Birthplace (Hong Kong)^a^	1.514	1.155–1.984
Gender (Male)^a^	0.402	0.306–0.529
History of Hypertension (With)^a^	0.440	0.337–0.575
History of Diabetes Mellitus (With)^a^	0.366	0.255–0.526
History of Hyperlipidemia (With)^a^	0.477	0.349–0.650
History of Heart Disease (With)	0.839	0.541–1.301
CVD Risk Factors Knowledge (Full)	1.322	0.995–1.757

^a^ indicates factors found to be statistical significant

## Discussion

Previous studies have clearly demonstrated that patient recognition of CVD risk factors was an important prerequisite to prevent CV incidents [24]. Therefore, it is hypothesized that recognizing CVD risk factors is an indicator of higher CV health awareness, and subsequent better CV health performance. While there are a lot of Caucasian data of people’s knowledge towards CV health and CVD risk factors, the situation in Hong Kong is yet to be addressed. To the best of our knowledge, this was the first study that focus the CV health and awareness of Hong Kong people.

Our study revealed that the prevalence of ideal CV health was extremely low among Hong Kong population which is similar to the studies in other countries, with a range of 0.1 to 1.0% [25–30]. However, there was a higher proportion of participants (35.9%) with five or more ideal metrics in Hong Kong than the study conducted by Folsom et al. (12.2%), Heart SCORE (less than 10%) and HONU Project (21.9%) [26–29], indicating that the CV health in Hong Kong may be better. It is however worthwhile noticing that the middle-aged and elderly groups perform equally worse than young adults, thus prompting the need for more resources for middle-aged outreach service in the future.

For individual health metrics, the study cohort of Hong Kong also performed better than all other studies in total cholesterol, BMI, smoking status, and diet but worse in blood glucose [26–30]. Furthermore, there is much room for improvement in physical activity in middle-aged group, blood pressure in elderly group and diet in younger adult group. These, together with blood glucose, are potential areas that warrant more focus in the planning of future cardiovascular health promotion campaigns.

It should, however, be noted that the ideal metrics in LS7 was defined by AHA in 2010 and has not updated since then. However, the treatment guidelines of different health indicators have been evolving. JNC 8 suggested initiating pharmacological treatment in the general population when systolic BP ≥140/90 mmHg (systolic BP > 150 mmHg for population aged ≥60) [31]. If we take this value as ideal BP, the percentage of subjects meeting the criteria would be doubled from 32.7% (*n* = 326) to 75.5% (*n* = 752). On the other hand, the development of LS7 and the definition of ideal metrics were based on the American population but have not been well studied in Asian population. Due to the differences between races, the cut-off of different health factors may vary. The definition of ideal BMI in LS7, for instance, is less than 25 kg/m^2^. However, it was proposed that a BMI of 23 kg/m^2^ or more would be a CVD risk factor in Asian population [32]. If we take 23 kg/m^2^ as a cut-off of ideal BMI, only 55.0% (*n* = 548) instead of 73.7% (*n* = 734) would meet the criteria. As a result, the actual CV health condition of the population may be under- or over-estimated.

In terms of LS7 score, younger and more educated subjects were more likely to perform better. Subjects born in Hong Kong were slightly more likely while male subjects are less likely to have optimal LS7. These results revealed the need of a CV health improvement programs targeting less educated male elders. To our surprise, no association was found between CVD risk factor knowledge and LS7 score. This implies people fail to execute healthy lifestyle behaviors even if they have adequate CV knowledge. The hinders for optimal LS7 should be explored so that future CV health programs could stress the importance of implementation rather than knowledge acquisition. On the other hand, patients with hypertension and diabetes also had lower LS7 scores, indicating the lack of self-awareness of the diseases prognosis. Despite the lack of negative direct proportion as seen in patients with hypertension and diabetes, patients with heart disease were also unlikely to optimize their cardiovascular risk. With the emphasis of preventive medicine in modern era, health education focusing on non-pharmacological management of different diseases should be promoted. Further research should be done to examine the potential methods further reduce their cardiovascular risks.

More than 30% of participants were able to identify all 11 CVD risk factors in the questionnaire and more than 70% of respondents recognized at least 9 of the risk factors. These results were consistent with a U.S. cross-sectional survey in which 37% of primary care patients was able to identify all 7 components of cardiac risk factors of myocardial infarction [14]. For individual components of CVD risk factors, our study revealed that obesity and family history were less recognized as CVD risk factors when compared to another U.S. cross-sectional study involving 172 18–26-year-old African American adults [33].

There were also certain limitations in our study. Selection bias could not be avoided due to the voluntary nature of outreach services in CUHK campus and elderly centers. As a result, there were a gender imbalance (72% of respondents being female) and education level difference in the current study and thus the results may not be generalized to the whole population in Hong Kong. The self-report nature of the current study would also stimulate a passive recall bias of CVD risk factors as they were stated explicitly in the questionnaire among other potential factors.

This pilot study has portrayed the local CV health on the basis of LS7, CVD risk knowledge background and offers a valuable glimpse into the CV health condition in Hong Kong. The results have provided baseline characteristics of CV health in Hong Kong, as well as shed light on the aspects of CVD risk factors that need more focus. Future studies can prospectively follow the CVD risk factor knowledge change for a period of time to see the trend of CVD risk perception in Hong Kong people. It is also meaningful to see if a graded relationship occurs between CV health and CVD incidence or mortality.

## Conclusion

The current study showed that the prevalence of ideal CV health is low in Hong Kong. Middle-aged and elderly subjects have worse CV health than young adults to a similar extent. More resources should be deployed to the less educated male elderly population that shows poorer CV health. CVD risk factor knowledge has no association while young age and tertiary education has positive association with CV health. The best recognized CVD risk factor is high blood pressure, whereas the least recognized ones are diabetes and family history.

## Additional file

Additional file 1: Questionnaire. (DOCX 25 kb)

## Data Availability

The datasets used and/or analyzed during the current study are available from the corresponding author on reasonable request.

## Abbreviations

AHA: American heart Associaton
BMI: Body mass index
CUHK: Chinese Univeristy of Hong Kong
CV: Cardiovascular
CVD: Cardiovascular diseases
LS7: Life’s simple 7
