# Supplementary material for: Life’s simple 7 and cardiovascular disease risk knowledge in Hong Kong
Source: BMC Cardiovasc Disord. 2019 Aug 2;19:185. doi: 10.1186/s12872-019-1171-7 (PMC6679482; doi:10.1186/s12872-019-1171-7)
Supplement: Supplementary file 1 — Questionnaire. (DOCX 25 kb) [file 12872_2019_1171_MOESM1_ESM.docx]

**Awareness and Knowledge**

Based on your knowledge about cardiovascular disease, which of the followings are risk factors of cardiovascular diseases or stroke?

|  | Agree | Neutral | Disagree | Don’t Know |
| --- | --- | --- | --- | --- |
| Female gender |  |  |  |  |
| Older than 40-year-old |  |  |  |  |
| Older than 65-year-old |  |  |  |  |
| High blood pressure |  |  |  |  |
| High blood sugar |  |  |  |  |
| High blood lipid |  |  |  |  |
| Smoking |  |  |  |  |
| Lack of exercise |  |  |  |  |
| More than 3 exercises per week |  |  |  |  |
| Less than 6-hour sleep per day |  |  |  |  |
| Daily vitamin supplements |  |  |  |  |
| Family history |  |  |  |  |
| Fish intake |  |  |  |  |
| Fruit or vegetable intake |  |  |  |  |
| High-salt diet |  |  |  |  |
| Sweetened drinks |  |  |  |  |
| Stress |  |  |  |  |
| More than 3 glasses of alcohol per week |  |  |  |  |

Do you agree that lifestyle modification can prevent cardiovascular disease and reduce the risk of heart attack?

| Agree | Neutral | Disagree | Don’t Know |
| --- | --- | --- | --- |

Which of the following activities are important to prevent heart attack?

|  | Agree | Neutral | Disagree | Don’t Know |
| --- | --- | --- | --- | --- |
| Exercise |  |  |  |  |
| Weight reduction |  |  |  |  |
| Stress reduction |  |  |  |  |
| Cholesterol intake reduction |  |  |  |  |
| Salt intake reduction |  |  |  |  |
| Smoking cessation |  |  |  |  |
| Using multivitamins |  |  |  |  |
| Using aspirin |  |  |  |  |

**Cardiovascular Risk-Lowering Action**

1. Do you exercise at least 20 minutes three times per week?

| Yes | No |
| --- | --- |

1. In the past one month, how long and how many times did you perform physical activities of moderate- (defined as those which will slightly speed up breathing and heart rates, and cause mild sweating but not so intense as to cause fatigue) to vigorous-intensity (defined as those which will greatly speed up breathing and heart rates and cause profuse sweating and fatigue)?

   Frequency: _______________
   Total time spent: _______________
2. Which of the following best describes your diet?

| Very Healthy | Healthy | Normal | Unhealthy | Very Unhealthy |
| --- | --- | --- | --- | --- |

1. How much and how many times did you eat/drink in the past 7 days?

| Food | Amount | Frequency |
| --- | --- | --- |
| Fruit |  |  |
| Vegetable |  |  |
| Fish |  |  |
| Meat |  |  |
| Whole-grain |  |  |
| Refined-grain |  |  |
| Sweetened beverages |  |  |
| Wine |  |  |

1. Which of the following best describes your salt consumption?

| Low | Medium | High |
| --- | --- | --- |

1. Which of the following best describes your oil consumption?

| Low | Medium | High |
| --- | --- | --- |

1. Were you involved in the following activities in the past 12 months
2. Which of the following channels would you obtain health-related information?

| Doctors | Nurses, pharmacists, physiotherapists or dietitians | Chinese medicine practitioners | Radio |
| --- | --- | --- | --- |
| TV | Friends/Family/Relative | Newspaper/Magazine | Internet |
| Health Seminars/Exhibitions | | Others |  |

1. Which of the following factor(s) may encourage you to adopt cardiovascular risk-lowering action? (can select more than one)

| Being healthier/longer life expectancy |
| --- |
| Stopping medications |
| For family |
| Understanding more about cardiovascular diseases |
| Advices from doctors |
| Relatives or friends dying from cardiovascular diseases |
| Encouragement from relatives or friends |
| Being diagnosed with cardiovascular diseases |
| Others: Please specify |

1. Which of the following factor(s) may hinder you from adopting cardiovascular risk-lowering action?

| Lack of time |
| --- |
| Low risk of cardiovascular diseases |
| Unwilling to modify current lifestyle |
| Financial burden for healthcare expenditure |
| Unclear actions that could be taken |
| Questionable efficacy for the risk-lowering action |
| My health being determined by God or other higher power |
| Afraid of changes |
| Anxiety/stress related issues |
| Actions too complicated |
| Needs to take care other family members |
| Lack of advices from doctors |
| Unclear advices from doctors |
| Others: Please specify |

1. Do you have health insurance?

| Yes | No |
| --- | --- |

**Background**

1. Which of the following best describes your health conditions?

| Excellent Health | Good Health | Acceptable | Poor |
| --- | --- | --- | --- |

1. In a scale of 0 – 100, with 100 being perfect health while 0 being death, how would you describe your health conditions?
   ________
2. Do you smoke?

| Yes – to Q5. | No – to Q4. |
| --- | --- |

1. Did you smoke?

| Yes – to Q5. | No – to Q6. |
| --- | --- |

1. Did you try to quit smoking in the past 12 months?

| Yes | No |
| --- | --- |

1. Were you diagnosed with heart disease?

| Yes – to Q7. | No – to Q8. |
| --- | --- |

1. What kind(s) of heart disease(s) was/were you diagnosed?

| Coronary Artery Disease (Heart Attack, Angina) | Heart Failure | Diseases involving heart valves | Arrhythmias | Congenital Heart Disease | Others | I don’t know |
| --- | --- | --- | --- | --- | --- | --- |

1. Do you have high blood pressure?

| Yes – to Q9. | No – to Q10. | I don’t know – to Q10. |
| --- | --- | --- |

1. Are you taking any drug(s) to lower your blood pressure?

| Yes | No |
| --- | --- |

1. Do you measure your blood pressure at home? If yes, how frequent do you measure your blood pressure?

| Yes, Always | Yes, Sometimes | Yes, Often | No |
| --- | --- | --- | --- |

1. Were you diagnosed as diabetes?

| Yes – to Q12. | No – to Q14. | I don’t know – to Q14. |
| --- | --- | --- |

1. Are you taking/using any drug(s) to lower your blood sugar?

| Yes | No |
| --- | --- |

1. Do you measure your blood sugar at home? If yes, how frequent do you measure your blood sugar?

| Yes, Always | Yes, Sometimes | Yes, Often | No |
| --- | --- | --- | --- |

1. Do you have high blood lipid?

| Yes – to Q15. | No – to Q16. | I don’t know – to Q16. |
| --- | --- | --- |

1. Are you taking/using any drug(s) to lower your blood lipid?

| Yes | No | I don’t know |
| --- | --- | --- |

1. Education Level:

| No formal education | Primary School | Secondary School | University or higher |
| --- | --- | --- | --- |

1. When were you born?
   _________
2. Family Income

| Government Subsidy | HKD  0 – 5000 | HKD  5,000 – 10,000 | HKD  10,000 – 30,000 | HKD 30,000 – 50,000 | HKD 50,000 or more | Don’t Know / Refuse to Answer |
| --- | --- | --- | --- | --- | --- | --- |

1. Where were you born?
   ___________________
2. Gender: M/F
